# Synthesis of curcumin-derived carbon dots with dual antimicrobial and osteogenic properties for enhanced bone regeneration

**DOI:** 10.3389/fcell.2026.1865318

**Published:** 2026-08-19

**Authors:** Yu Zhang, Lingyi Kong, Gang Dong, Qingqing Du

**Affiliations:** School of Stomatology, Qilu Medical University, Zibo, China

**Keywords:** antibacterial activity, bone defects, curcumin-derived carbon dots, osteogenic differentiation, rBMSCs

## Abstract

**Introduction and Aims:**

Infectious alveolar defects in dentistry present a persistent clinical dilemma, necessitating approaches that simultaneously promote osseous repair and suppress microbial adhesion. This study aimed to characterize the bifunctional properties of curcumin-derived carbon dots (Cur-CD) in facilitating the osteogenic commitment of rat bone marrow mesenchymal stem cells (rBMSCs) and exerting antibacterial effects, and offers foundational proof-of-concept data to support further evaluation in peri-implantitis-related infectious models.

**Methods:**

Cur-CD complex was synthesized and thoroughly characterized. Primary rBMSCs were isolated and cultured. The optimal working concentration of Cur-CD was identified via CCK-8 analysis. The cells were divided into control (Ctrl) and Cur-CD-treated groups. The degree of osteogenic differentiation was measured by Alizarin Red S staining, while protein and mRNA expression of key osteogenic markers (Runx2, OPN, Osterix, and OCN) were analyzed via Western blotting and quantitative real-time polymerase chain reaction, respectively. An oxidative stress model was established with H_2_O_2_, and cell migration was assessed by the transwell assay. The microbicidal properties against *Escherichia coli* and *Staphylococcus aureus* were examined using PI staining, plate colony counting, and inhibition zone assays.

**Results:**

The Cur-CD complex significantly enhanced mineralized nodule formation and upregulated both protein and mRNA expression of Runx2, OPN, Osterix, and OCN compared to the Ctrl group. Under H_2_O_2_-induced oxidative stress, Cur-CD treatment markedly promoted rBMSC migration. Additionally, the complex substantially increased bacterial mortality and reduced colony formation in *E. coli* and *S. aureus.*

**Clinical Relevance:**

These results demonstrate Cur-CD’s dual osteogenic and antibacterial capacity, laying groundwork for future exploration of its utility against peri-implantitis-associated bone defects.

**Conclusion:**

The Cur-CD complex demonstrates promising dual functionality by enhancing osteogenic differentiation of rBMSCs and exerting robust antibacterial effects.

## 1 Introduction

With the advancement of oral implantology, implant-supported prostheses have become the preferred treatment for patients with partial edentulism. However, the increasing incidence of peri-implantitis threatens long-term implant success. Peri-implantitis arises from multiple factors, including early loading, poor oral hygiene, and smoking (Smeets et al., 2014), although plaque accumulation remains the primary cause (Pranskunas et al., 2016). Clinical manifestations range from gingival redness and bleeding in mild cases to alveolar bone resorption in severe cases (Monje et al., 2019). Consequently, promoting peri-implant bone regeneration while achieving effective antibacterial outcomes is a core strategy for controlling this disorder (Klinge et al., 2018).

In osteogenesis and antimicrobial therapy, several single-agent compounds, such as taraxasterol, icariin, puerarin, and HU-308, have the potential to stimulate bone formation and inhibit microbial growth (Xiao et al., 2020; Zhang et al., 2021; Shaukat et al., 2022; Yang and Wang, 2024; Tian et al., 2025; Yang et al., 2025). However, monotherapy often fails to provide sustained drug release, limiting its clinical applicability. To address this issue, researchers have explored implant surface modifications and advanced biomaterials capable of delivering dual osteogenic and antibacterial effects. For example, silver-doped ceramic bioscaffolds exhibit excellent antibacterial performance (Xiao et al., 2020). Similarly, autologous biological carriers such as platelet-rich fibrin (PRF) and concentrated growth factor offer dual functionality: they serve as effective drug delivery systems while promoting osteoblast growth by releasing bioactive factors and nutrients (Ren et al., 2022). Previous studies have confirmed that puerarin-loaded PRF membranes significantly improve osteoblast activity. Electrospun polycaprolactone-based delivery systems have also exhibited promise in overcoming monotherapy limitations, despite challenges, such as incomplete biodegradation (Li and Tan, 2014; Preethi Soundarya et al., 2018). Consequently, identifying suitable drugs and developing high-performance carrier materials remain crucial.

Curcumin (Cur), the principal active constituent of turmeric, exhibits comprehensive biological functions, including antimicrobial, antiviral, and antiparasitic activities (Moghadamtousi et al., 2014; Praditya et al., 2019; Rai et al., 2020). In validated *in vitro* dynamic biofilm models, Cur extract demonstrated significant antibacterial efficacy against six bacterial species (Alonso-Espanol et al., 2023). Its safety, tolerability, and non-toxicity have been established in clinical trials (Gupta et al., 2013; Panknin et al., 2023). Furthermore, multiple studies have indicated that Cur can prevent bone loss caused by ovariectomy, elevate bone mineral density, alleviate osteoporosis and arthritis, and optimize bone microstructure (Khanizadeh et al., 2018; Yang et al., 2023).

Despite its attractive pharmacological properties, Cur has clinical limitations due to its poor water solubility, which impairs its bioavailability and bioactivity (Kotha and Luthria, 2019). Our research group has established expertise in carbon nanoparticle fabrication. In this study, we used Cur as a precursor to synthesize Cur-derived carbon dots (Cur-CD), effectively overcoming its poor water solubility and enhancing its functional performance. We aim to systematically investigate the osteogenic and antibacterial mechanisms of these Cur-CD, with the current rat calvarial defect model serving as a preliminary screening platform for subsequent infectious peri-implantitis research.

## 2 Materials and methods

### 2.1 Preparation of Cur-CD

First, 0.368 g Cur was dissolved in 10 mL of deionized water and then blended with 10 mL of silane coupling agent KH792. The mixture was transferred to a polytetrafluoroethylene-lined reactor and heated at 180 °C for 6 h, then allowed to cool naturally. The product was centrifuged at 5000 r/min for 10 min, and the supernatant was collected and washed thrice with cyclohexane to remove excess coupling agent. The aqueous phase containing Cur-CD was retained for further use.

### 2.2 Material characterization

The structural features of specimens were investigated using transmission electron microscopy (TEM). X-ray photoelectron spectroscopy (XPS) was utilized to determine the elemental composition, and Fourier transform infrared spectroscopy (FTIR) was applied to identify functional groups within 4000–400 cm^–1^. Surface charge and dispersion stability were assessed using zeta potential (ZP) and particle size measurements.

### 2.3 Cell isolation and culture

Marrow was isolated from the femora and tibiae of Sprague–Dawley (SD) rats with α-MEM containing 10% FBS. Following centrifugation, cells were maintained in complete growth medium at 37 °C under 5% CO_2_. Flow cytometry was used to verify cell phenotype, and subculturing was performed when cell density exceeded 90%.

### 2.4 Cell viability assay

Rat bone marrow mesenchymal stem cells (rBMSCs) were seeded into 96-well plates at a density of 2 × 10^3^ cells per well with 100 μL of growth medium. Following 24 h of incubation at 37 °C, the cultures were treated. After medium replacement with CCK-8 solution, plates were incubated for 2 h in the dark. The optical density at 450 nm was measured using a microplate reader.

For complementary biosafety evaluation, Annexin V-FITC/PI dual staining flow cytometry was performed across all Cur-CD concentration groups. Briefly, rBMSCs received gradient Cur-CD treatment for 24 h, harvested, resuspended in binding buffer, stained in darkness, and analyzed by flow cytometry to quantify viable and apoptotic cell populations.

### 2.5 Osteogenic differentiation assay

rBMSCs were inoculated into 6-well plates at a density of 5 × 10^4^ cells/mL and divided into control (Ctrl) and Cur-CD-treated groups. At 80% confluence, the medium was replaced with osteogenic induction medium (refreshed every 2–3 days), and the Ctrl group received standard medium. After 21 days, cells were fixed at room temperature for 20 min, washed with PBS, and stained with Alizarin Red S for 10 min. After washing, mineralized deposits were observed and photographed using an inverted microscope.

### 2.6 Transwell migration assay

rBMSCs were resuspended in serum-free medium at 2 × 10^5^ cells/mL and assigned to Ctrl and Cur-CD groups. A volume of 600 μL complete medium with or without Cur-CD was added to the lower Transwell chamber, and 200 μL cell suspension was loaded into the upper chamber. After 24 h of incubation, the stationary cells on the upper membrane were removed. Cells that migrated to the lower surface were fixed, stained with 0.1% crystal violet, photographed, and quantified.

### 2.7 Western blotting

Samples were lysed on ice using RIPA buffer for 30 min and centrifuged at 12,000× g (4 °C and 5 min). The supernatant was retained, and protein concentration was measured using the BCA assay. Equal protein loads were separated via SDS-PAGE and blotted onto PVDF membranes at 100 V for 1.5 h. After blocking with 5% skim milk, membranes were incubated with primary antibodies at 4 °C overnight. After TBST rinsing, horseradish peroxidase (HRP)-conjugated secondary antibodies were added for 1 h at room temperature. Protein bands were detected using ECL solution.

### 2.8 Quantitative real-time PCR (qRT-PCR)

RNA was extracted from cell samples using TRIzol lysis buffer. After cDNA preparation via reverse transcription, qRT-PCR was performed using SYBR Green and target-specific primers. Reactions were run on a qPCR instrument with standard thermal protocols, and all data were collected and analyzed.

### 2.9 DMAO/PI staining

*E. coli* and *S. aureus* were grown to log phase in LB broth at 37 °C. A 500 μL aliquot of each bacterial culture was mixed with an equal volume of Cur-CD or PBS and incubated at 37 °C for 4 h. After washing with PBS, bacteria were stained using a DMAO/PI staining kit, examined using fluorescence microscopy, and quantified.

### 2.10 Inhibition zone assay

*E. coli* or *S. aureus* suspensions were spread uniformly onto Mueller–Hinton agar plates. Sterile discs loaded with Cur-CD were placed on the agar surface. After 18 h of culture at 37 °C, the size of growth-inhibitory rings was determined.

### 2.11 RNA sequencing

rBMSCs were subjected to corresponding treatment with or without Cur-CD under osteogenic induction, with three independent biological replicates set for each experimental group (n = 3). After 21 days of osteogenic culture, total cellular RNA was extracted using TRIzol reagent according to the manufacturer’s instructions. RNA integrity and purity were strictly quantified to meet sequencing quality thresholds. Subsequently, mRNA was specifically captured and enriched from the total RNA using oligo (dT) magnetic beads. The enriched mRNA was randomly fragmented and reverse-transcribed into first-strand cDNA using random N6 primers, followed by second-strand cDNA synthesis. The resulting double-stranded cDNA fragments underwent end repair, 3′-adenylation, and ligation to unique Illumina sequencing adapters. Following PCR amplification and library quality control, the generated transcriptome libraries were subjected to high-throughput sequencing on the Illumina HiSeq platform using a 150 bp paired-end (PE150) strategy at Azenta Life Sciences. For subsequent bioinformatic analysis, raw sequencing data were first processed using Cutadapt (v1.9.1) to remove adapter sequences and low-quality bases, yielding high-quality clean reads. These clean reads were then aligned to the reference genome using HISAT2 (v2.2.1). Gene expression levels were calculated using HTSeq (v0.6.1) and normalized by the FPKM method. Differential gene expression analysis between the control and experimental groups was robustly performed utilizing the DESeq2 package (v1.26.0) based on the negative binomial distribution model, with stringent screening thresholds set at an adjusted P-value (padj) ≤0.05 and | log_2_ FoldChangege| ≥ 1. Finally, Gene Ontology (GO) and Kyoto Encyclopedia of Genes and Genomes (KEGG) enrichment analyses were carried out to dissect the transcriptional regulatory landscape and signaling pathways modulated by Cur-CD.

### 2.12 *In Vivo* calvarial defect model and evaluation

All animal protocols were approved by the Ethics Committee of Qilu Medical University and were conducted in compliance with institutional animal care and welfare regulations. Nine male SD rats weighing 250–300 g were randomly assigned to three groups (n = 3): Ctrl, Cur, and Cur-CD groups. Pentobarbital sodium (40 mg/kg) was administered intraperitoneally to anesthetize the animals. Bilateral 5-mm critical-size defects were generated in the rat parietal bones using a trephine bur, accompanied by constant saline perfusion to cool the surgical site. Bilateral critical-sized calvarial defects (5 mm in diameter) were created in the parietal bones using a trephine bur under continuous saline irrigation. After defect creation, different treatments were applied locally. Defects in the Ctrl group were filled with PBS, those in the Cur group received Cur, and those in the Cur-CD group were treated with Cur-CD composites. The periosteum and skin were then sutured layer by layer. Postoperatively, all animals received intramuscular antibiotics for 3 consecutive days to prevent infection and were maintained under standard conditions.

Four weeks after the operation, the rats were sacrificed, and skull samples were collected and fixed in 4% paraformaldehyde. Micro-CT scanning was used to assess bone repair in the defect region. Three-dimensional reconstruction was performed, and bone morphometric parameters, including bone volume fraction (BV/TV), trabecular thickness (Tb.Th), and trabecular number (Tb.N), were quantified. Following micro-CT scanning, the specimens were decalcified in 10% EDTA for 8 weeks, dehydrated through graded ethanol, embedded in paraffin, and cut into 5-μm sections. Hematoxylin and eosin (H&E) and Masson’s trichrome staining were applied to examine tissue structure, bone regeneration, and collagen deposition. Representative images were captured under a microscope, and ImageJ was used to measure the area of new bone.

To further evaluate osteogenic protein expression, immunohistochemistry (IHC) and immunofluorescence (IF) staining were performed. OPN expression was detected using IHC. Tissue sections were first deparaffinized and rehydrated, followed by pepsin-mediated antigen retrieval and blocked with 5% bovine serum albumin. Slides were then incubated at 4 °C overnight with OPN primary antibody (ab283656, Abcam, 1:200) and subsequently treated with HRP-conjugated secondary antibody (ab205718, Abcam, 1:500) at room temperature. Immunoreactive signals were detected using a DAB chromogenic kit, and hematoxylin was used for nuclear counterstaining. For IF analysis of OCN and Runx2, sections underwent similar antigen retrieval and blocking steps, and were incubated overnight at 4 °C with OCN (PA5-96529, Thermo Fisher Scientific, 1:200) and Runx2 (PA5-82787, Thermo Fisher Scientific, 1:200) primary antibodies. The following day, fluorescently labeled secondary antibody (Goat anti-Rabbit IgG (H + L), A-11008, Thermo Fisher Scientific) was applied under light-protected conditions, and nuclei were counterstained with DAPI. All images were acquired using fluorescence microscopy. All images were acquired using identical parameters. Three random fields were selected for analysis for each sample. Quantification was performed independently by two blinded investigators. Positive staining regions were quantified using ImageJ and expressed as a proportion of the total tissue area.

### 2.13 Statistical analysis

Statistical analyses were performed using GraphPad Prism software (version 10.0). Intergroup comparisons were conducted using independent-sample t-tests, while multigroup differences were evaluated using two-way analysis of variance. The results are expressed as mean ± standard deviation (SD). Statistical significance was set at *p < 0.05, **p < 0.01, and ***p < 0.001.

## 3 Results

### 3.1 Material characterization

**FIGURE 1:**
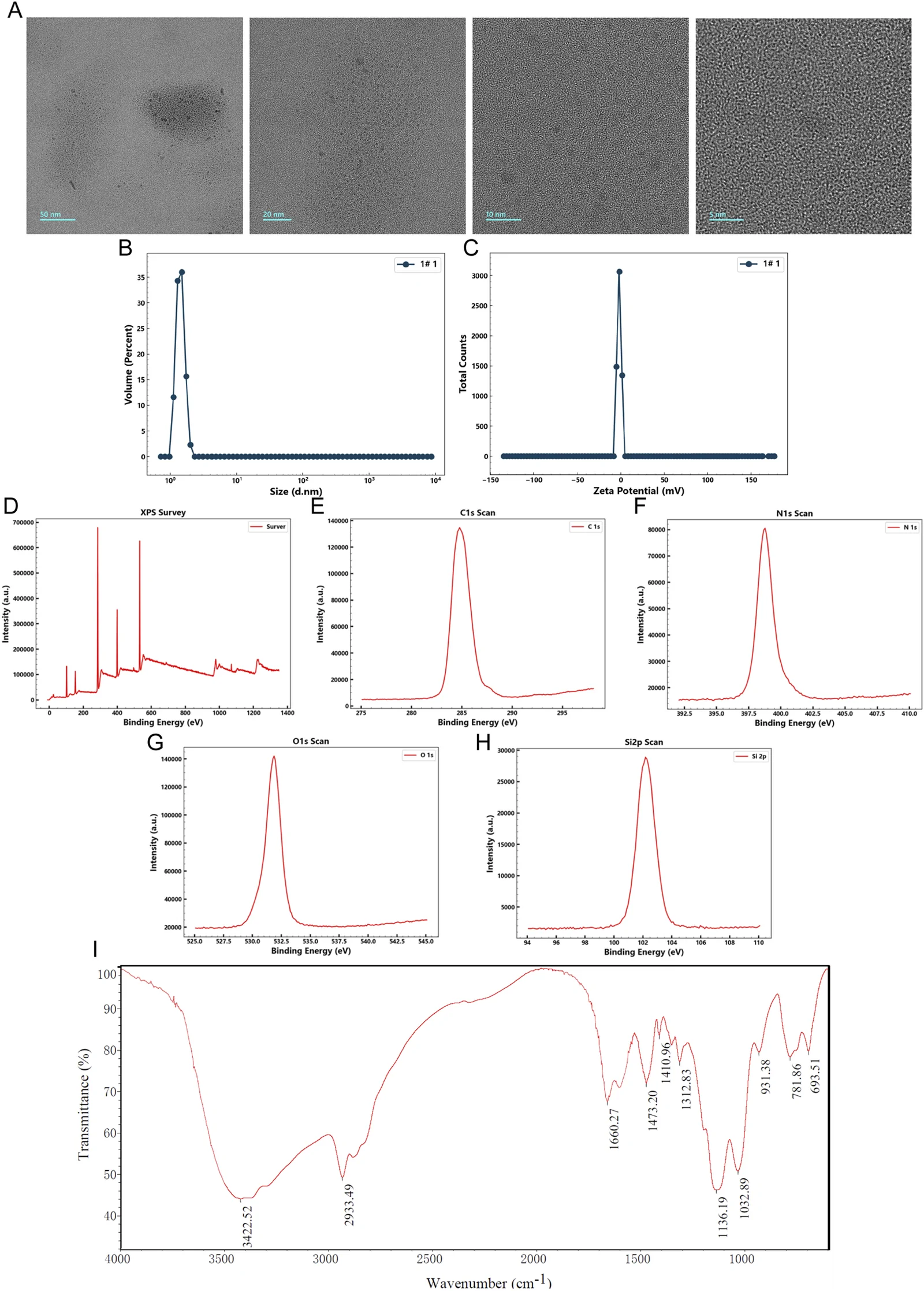
Characterization of the Cur-CD composite. **(A)** TEM images of the Cur-CD composite at different scales. **(B)** Particle size distribution analysis of the Cur-CD composite. **(C)** ZP analysis of the Cur-CD composite. **(D)** XPS survey spectrum. High-resolution XPS spectra of **(E)** C 1s, **(F)** N 1s, **(G)** O 1s, and **(H)** Si2p. **(I)** FTIR spectrum of the Cur-CD composite.

TEM was employed to elucidate the microstructure, particle size, and uniformity of the Cur-CD composite. Observations demonstrated that the Cur-CD complex displayed a spherical shape with consistent dimensions and homogeneous structure (Figure 1A). The particle size was predominantly within the range of 1.499 ± 0.4 nm (Figure 1B). ZP measurements, used to determine the surface charge, indicated a ZP value of -2.03 mV for the sample (Figure 1C). XPS was used to determine the surface elemental components and chemical valence states of the nanocomposite. As illustrated in the survey spectrum (Figures 1D–H), characteristic peaks confirmed the presence of C, N, O, and Si. The high-resolution C 1s spectrum (Figure 1E) exhibits a peak at 286 eV, indicating that carbon primarily exists in the sp^2^ hybridization state. The N 1s spectrum (Figure 1F) displays a peak centered at 399.3 eV, which is attributable to pyridinic/pyrrolic N within heterocyclic structures. The main peak in the O 1s spectrum (Figure 1G) was observed at approximately 532.5 eV, suggesting the presence of oxygen, predominantly as surface hydroxyl groups. The Si 2p spectrum (Figure 1H) exhibited a primary peak at approximately 102.5 eV, consistent with silicon existing in the form of SiO_2_. The functional moieties of the samples were examined using FTIR. As illustrated in Figure 1I, various characteristic absorption bands were observed in the 4000–400 cm^–1^ range. A wide absorption peak at 3422.52 cm^–1^ was attributed to O-H stretching vibrations, suggesting the existence of hydroxyl moieties derived from surface-adsorbed water or hydroxyl functional groups. The peak at 2933.49 cm^–1^ corresponds to the stretching vibrations of saturated C-H bonds, confirming the existence of aliphatic hydrocarbon structures. The peak observed at 1660.27 cm^–1^ was assigned to C=C stretching modes, suggesting the presence of unsaturated carbon-carbon bonds (such as those in aromatic rings or alkene structures). Peaks at 1473.20 cm^-1^ and 1410.96 cm^–1^, associated with C-H bending vibrations, provided further evidence of aliphatic or aromatic hydrocarbon structures. Strong absorption peaks at 1136.19 and 1032.89 cm^–1^ were assigned to the stretching vibrations of C-O-C ether bonds or C-OH alcoholic hydroxyl groups, indicating the presence of ether or alcohol functionalities. Characteristic peaks at 931.38, 781.86, and 693.51 cm^–1^ were related to the C-H out-of-plane bending vibrations of aromatic rings, confirming the existence of aromatic structures; the multiple peaks were consistent with the IR absorption behavior of monosubstituted benzene rings.

Overall, the material characterization results demonstrate that the Cur-CD composite consists of spherical particles with uniform sizes and structures. The analyses confirmed the co-existence of hydroxyl groups, aliphatic hydrocarbon structures, unsaturated carbon-carbon bonds, ether/alcohol functionalities, and aromatic structures within the sample, providing key spectroscopic evidence for its chemical composition and molecular structure.

### 3.2 Cur-CD complexes promote osteogenic differentiation of rBMSCs

**FIGURE 2:**
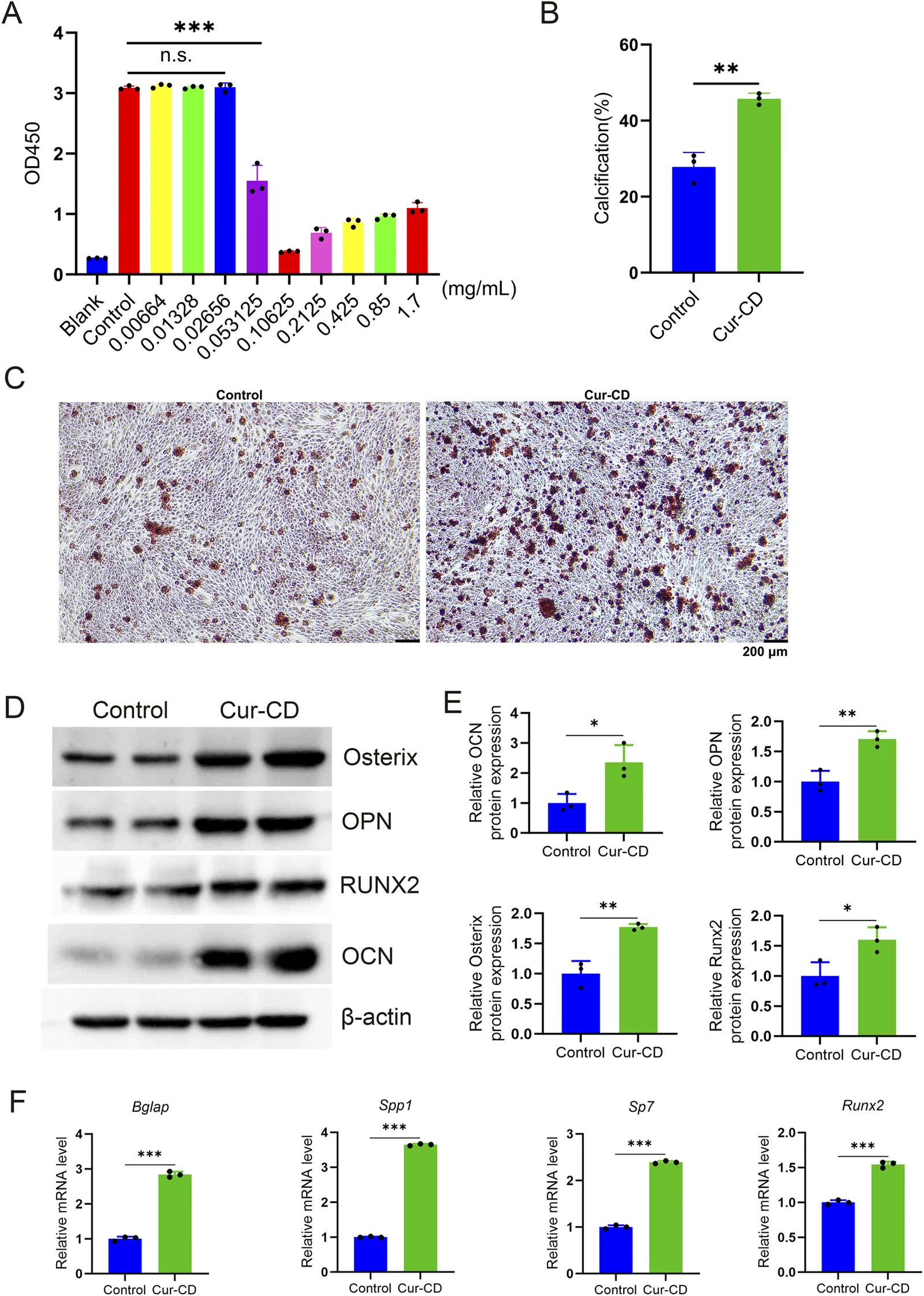
Effect of Cur-CD on osteogenic differentiation of rBMSCs. **(A)** Cell viability was assessed using a CCK-8 assay. **(B)** Quantitative analysis of mineralized nodule formation. **(C)** ARS staining. Scale bar = 200 μm. **(D)** Western blotting of Runx2, OPN, Osterix, and OCN protein expression levels. **(E)** Statistical results corresponding to **(D)**. **(F)** qRT-PCR analysis of Runx2, OPN, Osterix, and OCN mRNA levels.

To explore the influence of Cur-CD on the osteogenic differentiation of rBMSCs, cells were first exposed to gradient concentrations of Cur-CD. A CCK-8 assay was performed to evaluate the cytotoxic effects of Cur-CD on cellular activity. The results indicated that the maximum cell death ratio occurred at 0.10625 mg/mL, while no obvious cytotoxicity was detected at 0.02656 mg/mL (Figure 2A). Given that CCK-8 only reflects cellular metabolic status and cannot directly quantify apoptotic cell populations, we further implemented Annexin V-FITC/PI dual staining flow cytometry covering all tested Cur-CD concentrations. The flow cytometric dot plots intuitively corroborated the CCK-8 outcomes, with negligible apoptosis observed at 0.02656 mg/mL and severe apoptotic responses triggered at high concentrations (Supplementary Figure S1). Accordingly, this concentration was chosen for the subsequent experiments. After 21 days of osteogenic induction culture, Alizarin Red S staining was performed to assess matrix mineralization. The treated group displayed markedly more mineralized nodules than the Ctrl group, verifying that Cur-CD effectively promoted osteogenic differentiation in rBMSCs (Figure 2B-C; Supplementary Figure S2). Furthermore, the protein and transcript levels of core osteogenic regulators, including Runx2, OPN, Osterix, and OCN, were detected. Both protein expression (Figures 2D,E) and mRNA transcription (Figure 2F) of these markers were notably upregulated in the Cur-CD group compared to the Ctrl. These findings provide further evidence that the Cur-CD conjugate promotes the osteogenic differentiation of rBMSCs.

### 3.3 Cur-CD attenuates H_2_O_2_-induced impairment of rBMSC migration

**FIGURE 3:**
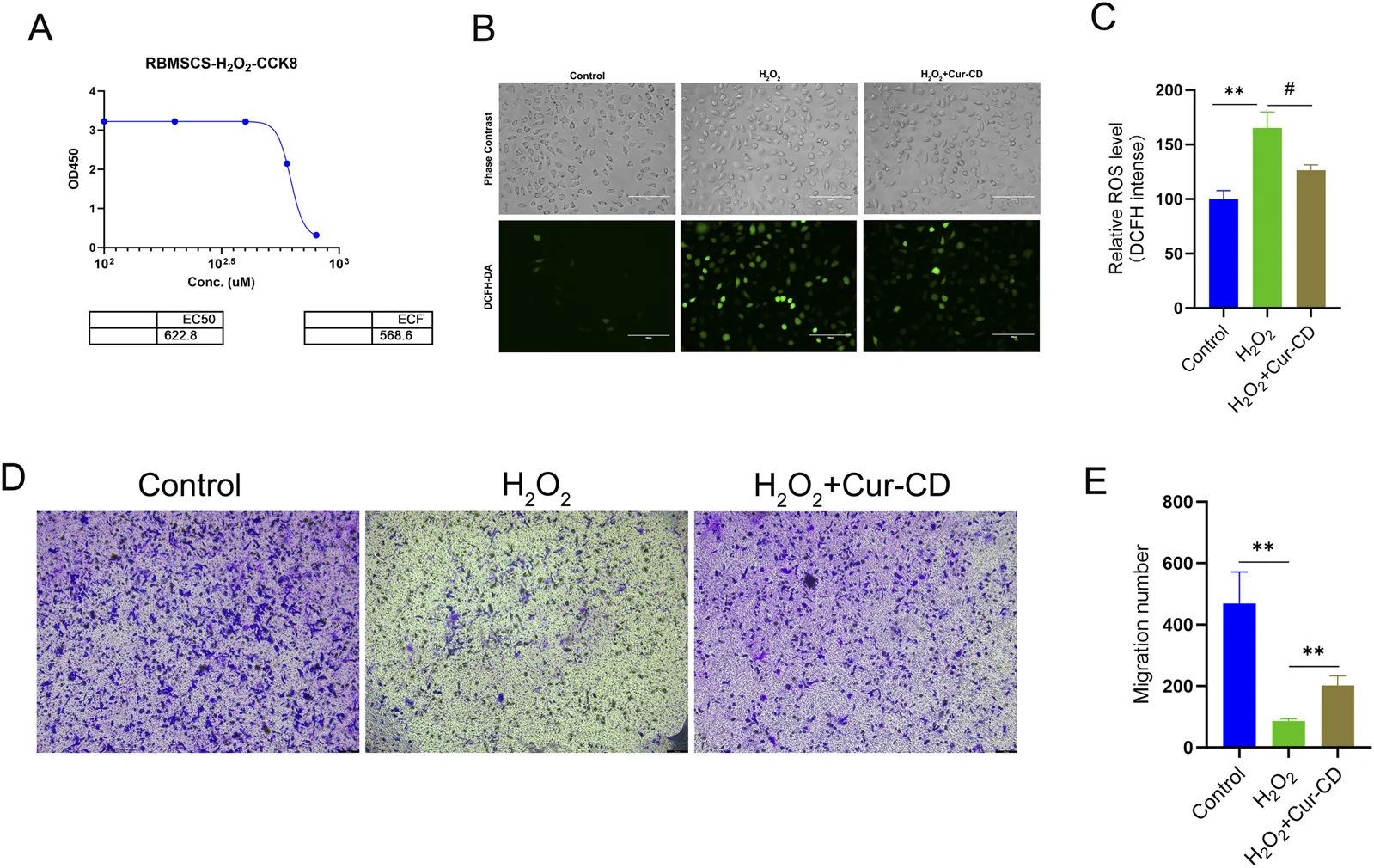
Cur-CD mitigated H2O2-impaired migration in rBMSCs by reducing oxidative stress. **(A)** Cell viability after H_2_O_2_ treatment (CCK-8 assay). **(B)** ROS fluorescence images (DCFH-DA staining). **(C)** Quantitative ROS levels. **(D)** Cell migration (wound healing assay). **(E)** Migration rate quantification.

To establish an *in vitro* oxidative stress model, rBMSCs were treated with H_2_O_2_ at concentrations of 0, 100, 200, 400, 600, and 800 μM for 24 h. The CCK-8 assay was used to evaluate cell viability and screen for the optimal H_2_O_2_ concentration. It was found that 600 μM H_2_O_2_ exhibited significant cytotoxicity relative to the Ctrl; therefore, this concentration was used in subsequent studies (Figure 3A). To assess the effect of H_2_O_2_ on intracellular reactive oxygen species (ROS) levels, rBMSCs were exposed to H_2_O_2_ and stained with the fluorescent probe DCFH-DA. Fluorescence imaging revealed a pronounced increase in ROS production after H_2_O_2_ treatment (Figure 3B). Quantitative analysis further demonstrated that Cur-CD treatment markedly reduced the H_2_O_2_-induced ROS elevation (Figure 3C). Next, we investigated whether Cur-CD could ameliorate the H_2_O_2_-impaired migratory capacity of rBMSCs. Scratch wound assays demonstrated that H_2_O_2_ markedly inhibited cellular migration, whereas Cur-CD intervention effectively restored this functional impairment (Figures 3D,E).

### 3.4 Cur-CD has significant antibacterial effect

**FIGURE 4:**
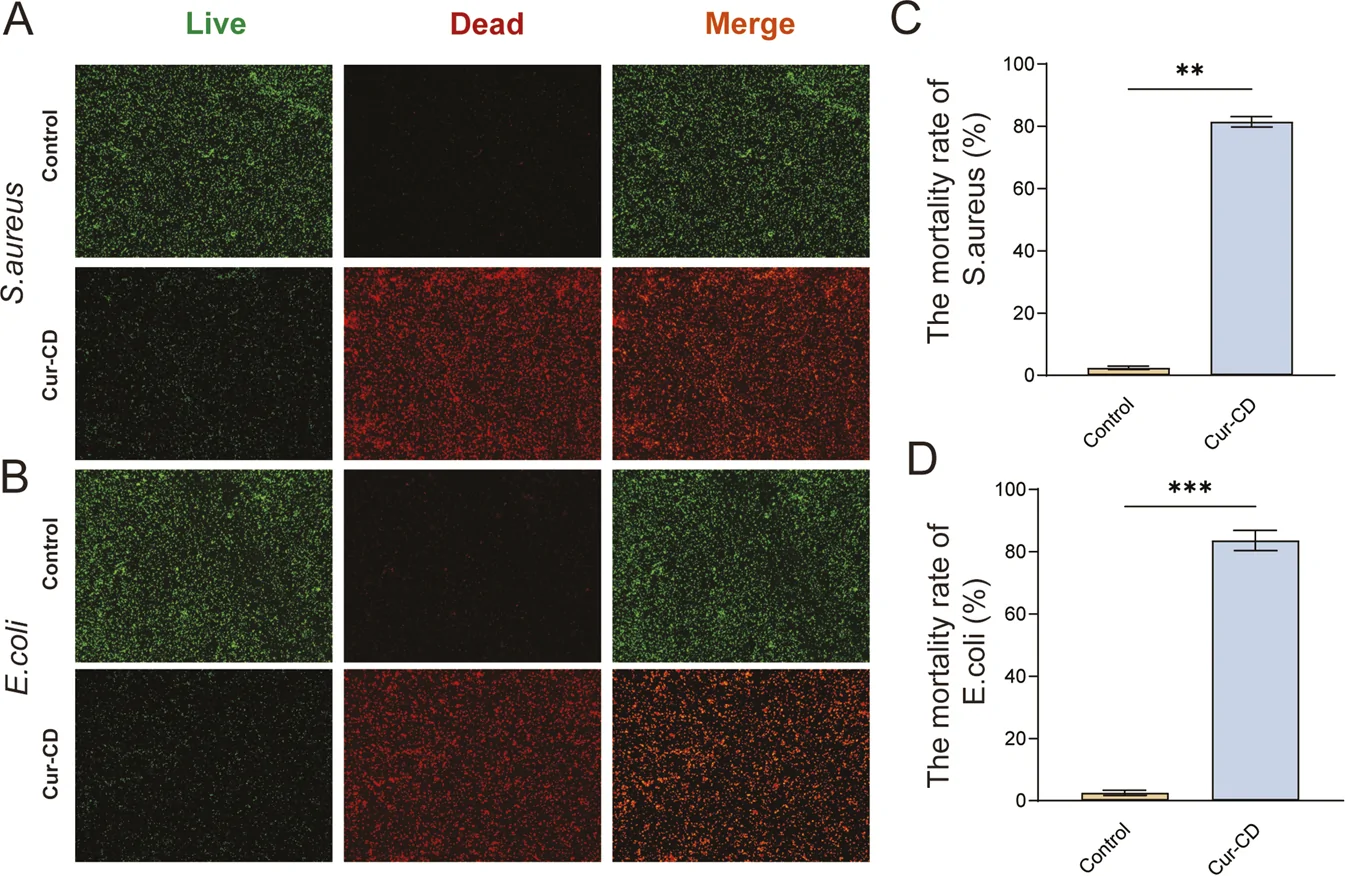
Cur-CD exhibited potent antibacterial activity against S. aureus and E. coli. **(A,B)** Representative fluorescence images of bacteria: live bacteria (green, DMAO staining) and dead or membrane-compromised bacteria (red, PI staining). **(C,D)** Quantitative analysis of PI fluorescence intensity, indicating bacterial mortality. Scale bar = 150 μm.

**FIGURE 5:**
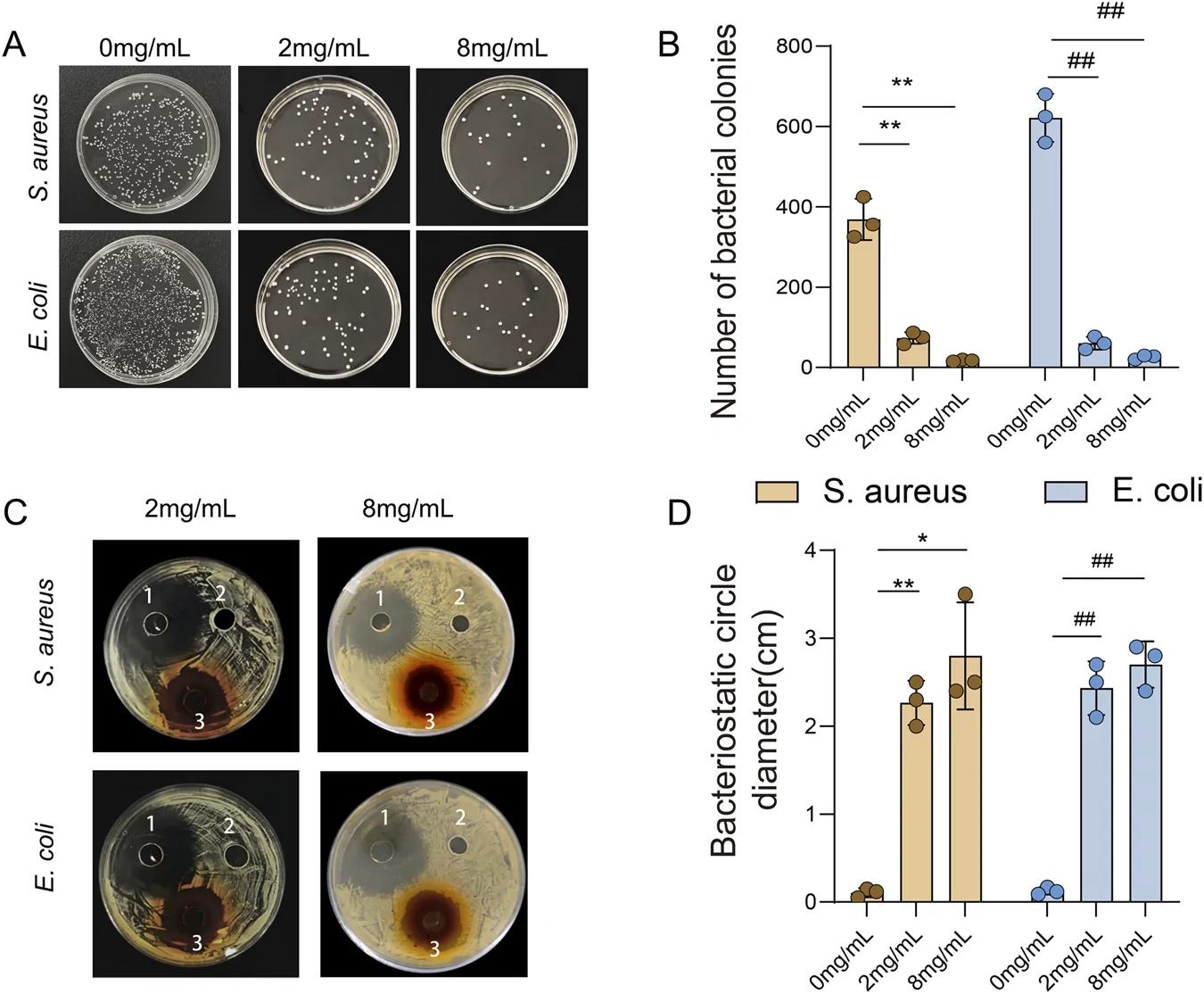
Evaluation of antibacterial efficacy of Cur-CD against S. aureus and E. coli. **(A)** Representative images of the spread plate assay exhibiting inhibition of bacterial growth by Cur-CD. **(B)** Quantitative analysis of colony-forming units. **(C)** Zone of inhibition assay. Discs: 1 = ampicillin (positive Ctrl), 2 = sterile water (negative Ctrl), 3 = Cur-CD. **(D)** Quantitative measurement of inhibition zone diameters.

To investigate the antibacterial capacity of the Cur-CD, *S. aureus* and *E. coli* were subjected to PI staining. Results exhibited that Cur-CD treatment led to a prominent increase in PI fluorescence intensity compared to the untreated group, indicating that Cur-CD treatment markedly increased the mortality rates of *S. aureus* and *E. coli* (Figures 4A–D). Moreover, the spread plate and inhibition zone assays indicated that Cur-CD significantly inhibited the proliferation of *E. coli* and *S. aureus* relative to Ctrl samples (Figures 5A–D). Overall, these results confirmed the strong antibacterial activity of the synthesized Cur-CD complex.

### 3.5 Transcriptome profiling reveals osteogenic differentiation pathways in rBMSCs treated with Cur-CD

**FIGURE 6:**
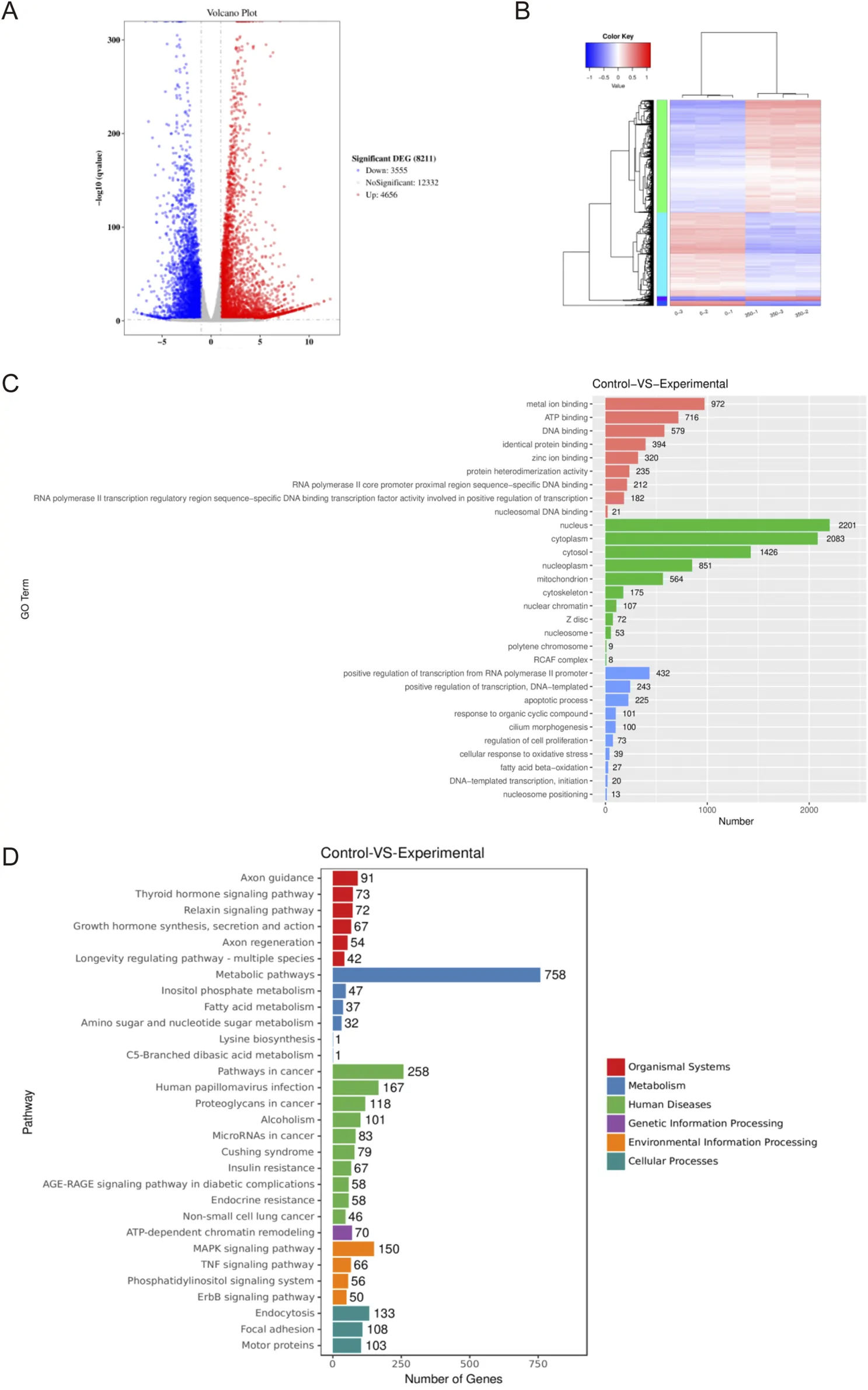
Transcriptomic profiling of rBMSCs treated with Cur-CD. **(A)** Volcano plot depicting differentially expressed genes (DEGs) between Ctrl and Cur-CD-treated groups. Red and blue dots denote significantly upregulated and downregulated genes, respectively. **(B)** Heatmap displaying expression patterns of DEGs across samples. **(C)** GO enrichment analysis exhibiting significantly enriched terms in biological process, molecular function, and cellular component categories. **(D)** KEGG pathway enrichment analysis of DEGs.

To investigate the underlying mechanisms of Cur-CD in osteogenic differentiation, rBMSCs from different experimental groups were subjected to 21 days of osteogenic induction, followed by total RNA extraction and sequencing. Transcriptome analysis identified 4,656 upregulated and 3,555 downregulated genes (Figures 6A,B). Gene ontology (GO) analysis revealed prominent enrichment in biological pathways associated with bone formation, extracellular matrix organization, and collagen fibril assembly. Kyoto encyclopedia of genes and genomes (KEGG) pathway analysis further identified key signaling pathways involved in osteogenesis, including TGF-β, Wnt, and MAPK signaling pathways (Figures 6C,D). Collectively, these results suggest that Cur-CD promotes osteogenic differentiation by modulating genes involved in matrix deposition and bone-related signaling cascades.

### 3.6 Cur-CD promotes bone regeneration and osteogenic activity *in vivo*

**FIGURE 7:**
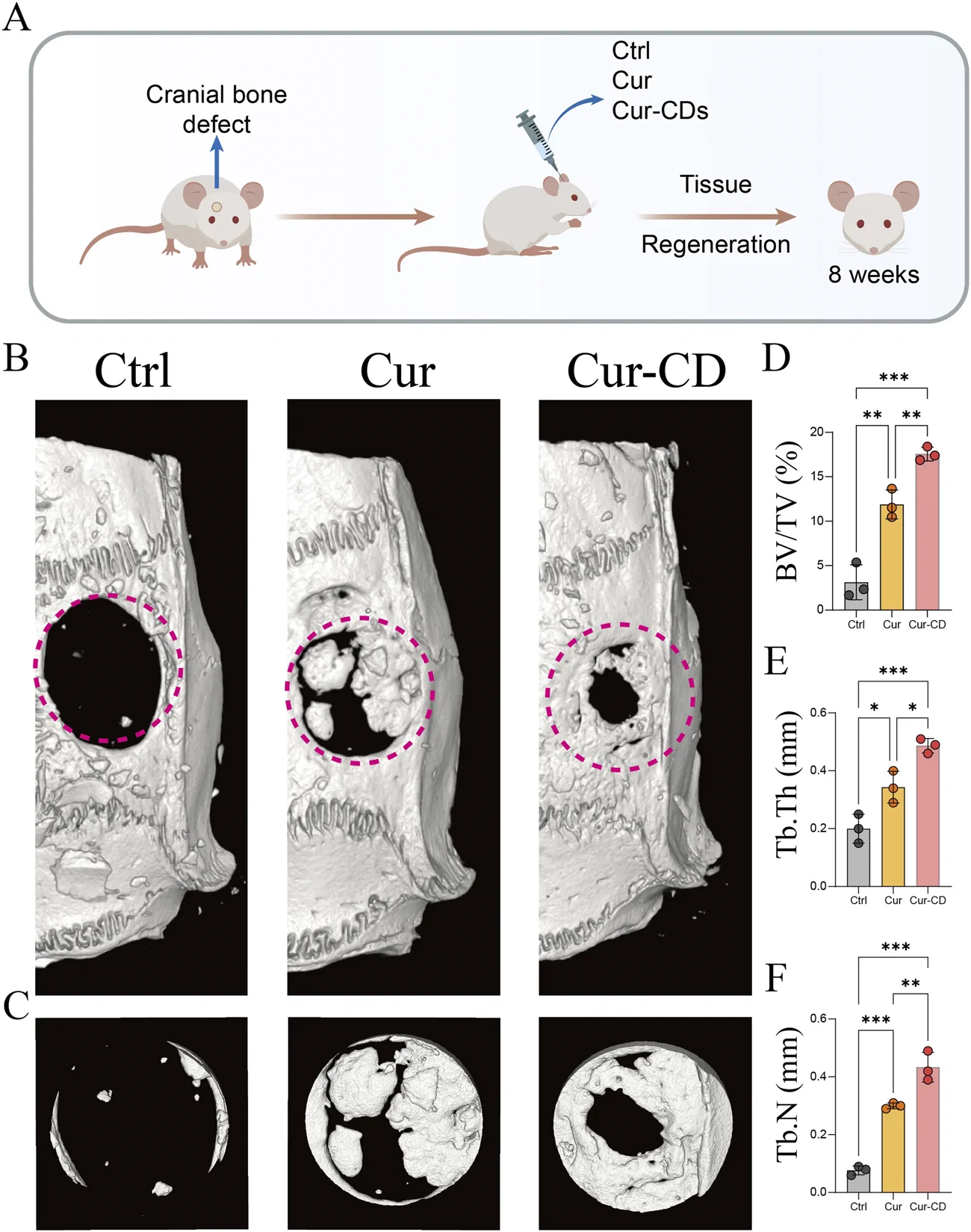
Evaluation of bone regeneration via micro-CT in rat cranial defects treated with Cur-CD. **(A)** Illustration of the experimental design and generation of the calvarial defect model. **(B,C)** Representative three-dimensional micro-CT reconstructions of the defect area and corresponding enlarged views. The dashed circles indicate the defect regions. **(D–F)** Quantitative analysis of bone morphometric parameters, including BV/TV, Tb.Th, and Tb.N. Data are presented as mean ± SD (n = 3). *p < 0.05, **p < 0.01, and ***p < 0.001.

To investigate the *in vivo* bone regenerative capacity of Cur-CD, micro-CT was first used to perform three-dimensional reconstruction and quantitative analysis of a rat calvarial defect model (Figures 7A-C). The reconstructed images revealed that the defect area in the Ctrl group remained largely unhealed, with only sparse and scattered new bone formation. In the Cur group, a moderate degree of new bone formation was observed; however, the defect was not sufficiently filled. Conversely, the Cur-CD group exhibited markedly enhanced bone regeneration, with newly formed bone extending progressively from the defect margins toward the center, and partial closure observed in some regions. These findings were consistent with quantitative morphometric analysis. The BV/TV value in the Ctrl group was 3.12% ± 1.97%, whereas it increased significantly to 17.55% ± 0.77% in the Cur-CD group (*p* < 0.001) (Figure 7D). Tb.Th and Tb.N reached their highest levels in the Cur-CD group (Figures 7E,F). Collectively, these results indicate that Cur-CD not only promotes new bone formation but also improves trabecular microarchitecture to a certain extent. Histological analyses were conducted to further corroborate the imaging findings.

**FIGURE 8:**
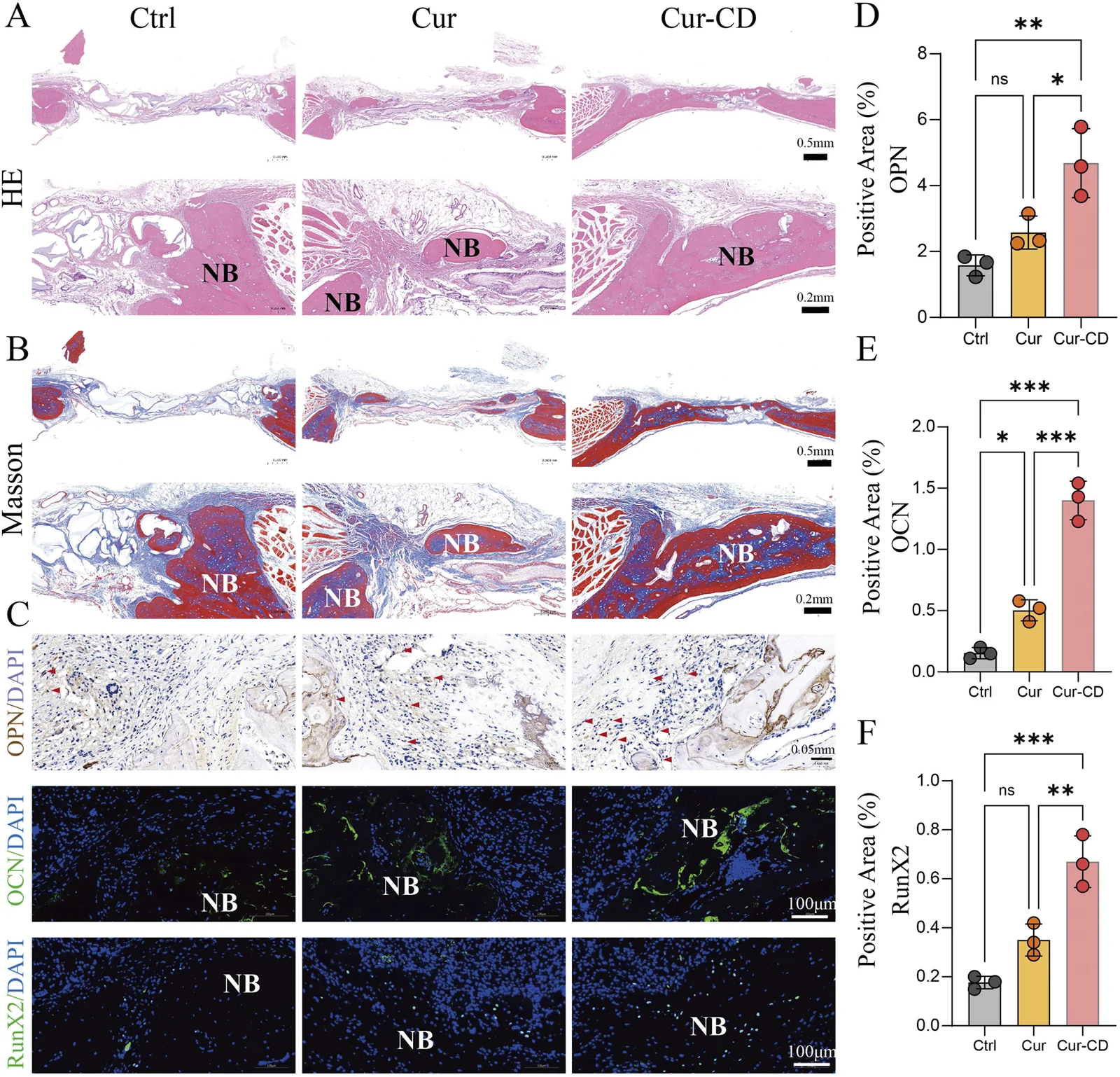
Histological and immunostaining analyses of bone regeneration in rat calvarial defects. **(A)** H&E staining. **(B)** Masson’s trichrome staining (blue: collagen fibers; red: mineralized bone). **(C)** Immunohistochemical and IF staining for osteogenic markers. OPN expression was detected by IHC (brown), while OCN and Runx2 were analyzed by IF (green), with nuclei counterstained with DAPI (blue). **(D–F)** Quantitative analysis of the positive staining areas of OPN, OCN, and Runx2. Data are presented as mean ± SD (n = 3). *p < 0.05, **p < 0.01, and ***p < 0.001; ns, not significant.

H&E staining revealed that the defect region in the Ctrl group was mainly occupied by fibrous connective tissue, with minimal new bone formation (Figure 8A). The Cur group displayed increased new bone formation, although the distribution was discontinuous. Conversely, abundant, more compact bone tissue was observed in the Cur-CD group, with bone-bridge formation evident in some areas. Furthermore, Masson’s trichrome staining demonstrated that collagen deposition in the Ctrl group was limited, moderately enhanced in the Cur group, and markedly increased collagen deposition accompanied by more mineralized bone tissue in the Cur-CD group (Figure 8B).

Further evaluation of osteogenesis-related protein expression was performed using IHC and IF analyses. Immunohistochemical staining revealed that the OPN-positive area increased from 1.58% ± 0.31% in the Ctrl group to 2.58% ± 0.49% in the Cur group, and further to 4.68% ± 1.05% in the Cur-CD group, which was notably higher than the corresponding level in the Cur group (*p* < 0.01) (Figures 8C,D). IF results revealed that the expression patterns of Runx2 and OCN were consistent with those of OPN, both peaking in the Cur-CD group. Relative to the Ctrl group, the Cur-CD group displayed prominently elevated Runx2-and OCN-positive regions, which were also higher than the Cur group (Figures 8E,F). Collectively, these findings demonstrate that Cur-CD significantly enhances the expression of osteogenesis-related proteins, thereby promoting osteogenic differentiation and bone matrix mineralization, ultimately leading to improved bone regeneration *in vivo*.

## 4 Discussion

Peri-implantitis is a common complication of oral implant rehabilitation that significantly compromises the long-term survival of dental implants (Noelken and Al-Nawas, 2023). Peri-implantitis occurs in as many as 4.7%–45% of patients and 3.6%–22.1% of implants, and is a bacterial-induced chronic inflammatory process that promotes osteoclast-mediated bone resorption and inhibits bone formation, leading to progressive bone loss around the implant (Wada et al., 2021). Consequently, the cornerstone of peri-implantitis treatment is promoting bone regeneration and inhibiting bacterial proliferation.

Cur, a natural polyphenol compound derived from turmeric, has demonstrated extensive therapeutic potential, including notable anti-inflammatory, anticancer, and anti-aging properties (Gupta et al., 2013; Tomeh et al., 2019; Liu et al., 2022; Yaikwawong et al., 2024). Particularly relevant to bone metabolism, Cur has exhibited significant inhibitory effects on osteoporotic processes by modulating densitometric indices and bone resorption markers (Lavhale et al., 2025). These biological effects are mechanistically linked to Toll-like receptor signaling and subsequent regulation of downstream pathways, including NF-κB, MAPK, and AP-1 signaling (Rahimifard et al., 2017; Zhang et al., 2019). Extensive research using inflammatory cell models and animal experiments has consistently demonstrated that Cur significantly reduces levels of various pro-inflammatory mediators, including Interleukin-1 (IL-1), IL-1β, IL-6, IL-8, IL-17, IL-27, tumor necrosis factor-α, inducible nitric oxide synthase, NO, tegulated upon activation normal T cell expressed and secreted factor, granulocyte colony-stimulating factor, and monocyte chemotactic protein-1 (Meng et al., 2013; Zeng et al., 2013; Sadeghi et al., 2018). Further evidence from clinical trials has verified these anti-inflammatory properties (Panahi et al., 2016).

Despite these promising properties, the clinical application of Cur remains largely limited because of its poor water solubility and rapid metabolic degradation (Feng et al., 2017). Extensive published evidence distinguishes the functional performance of free curcumin, bare carbon dots and integrated Cur-CD composites (Mathur et al., 2025; Arvapalli et al., 2020; Jian et al., 2023; Zhang et al., 2025). Free curcumin suffers from severe hydrophobicity and fast oxidative degradation under aqueous culture conditions, leading to insufficient cellular uptake and attenuated osteogenic/antibacterial effects. Bare carbon dots only possess weak intrinsic antioxidant capacity and lack potent osteoinductive and bactericidal activity. By contrast, the Cur-CD hybrid overcomes the solubility bottleneck of free curcumin and integrates the delivery advantages of carbon nanostructures with the robust bioactivity of curcumin, thereby generating synergistic therapeutic effects unattainable by either single component alone. To address these limitations, we developed a novel Cur-CD that demonstrates significantly improved physicochemical properties while maintaining and enhancing the biological activities of Cur. Regarding the intracellular action mode of Cur-CD, consistent with established carbon dot-curcumin composite paradigms (Mathur et al., 2025), ultrasmall carbon quantum dots enter mesenchymal stem cells via intact endocytosis. Ester linkages anchoring curcumin moieties to carbon scaffolds remain stable under neutral physiological conditions, while acid-triggered ester hydrolysis within endolysosomes enables sustained curcumin liberation without burst release.

This study systematically elucidated the osteogenic and antibacterial properties of this innovative composite, establishing a clear progression from molecular interactions to phenotypic outcomes.

Our findings demonstrate that Cur-CD significantly enhances osteogenic differentiation of rBMSCs. Alizarin Red S staining revealed that Cur-CD treatment promoted early osteogenic activity and substantially increased mineralized nodule formation compared to the Ctrl group. Furthermore, Western blotting and qPCR analyses confirmed marked upregulation of key osteogenic markers, including Runx2, OPN, Osterix, and OCN, following Cur-CD treatment. Runx2 and Osterix function as master transcription factors regulating osteoblast differentiation, whereas OPN and OCN play crucial roles in extracellular matrix mineralization. We speculate that Cur-CD not only delivers the bioactivity of Cur but that the nanostructured CD may also synergistically enhance osteogenic signaling by improving cell adhesion and facilitating cellular uptake.

In addition to its osteogenic effects, Transwell migration assays demonstrated that Cur-CD effectively enhanced the migratory capacity of rBMSCs. This finding is particularly relevant for bone regeneration applications, as efficient recruitment of mesenchymal stem cells to defect sites is the initial critical step in bone repair. The observed enhancement in directed cell migration with Cur-CD treatment suggests that the composite may upregulate signaling pathways involved in cell motility.

At the molecular level, transcriptome profiling revealed that Cur-CD treatment modulates critical signaling pathways essential for osteogenesis. Specifically, the TGF-β signaling cascade was activated, a pathway central to osteogenic differentiation and extracellular bone matrix formation. Concurrently, the Wnt signaling pathway, fundamental to bone development and homeostasis, was significantly enriched, suggesting coordinated regulation of multiple osteogenic cascades. Furthermore, the MAPK pathway, which is closely associated with cellular proliferation and differentiation, was activated upon Cur-CD administration.

Structural analysis provides additional mechanistic insights into these effects. FTIR characterization confirmed the presence of hydroxyl groups in Cur-CD, which likely facilitate interactions with cell-surface receptors and subsequent activation of osteogenic transcription factors. Additionally, the sp^2^-hybridized carbon framework contributes to both structural stability and bioactivity, creating a favorable microenvironment for osteogenic differentiation.

Regarding antibacterial performance, Cur-CD exhibited robust antibacterial activity against Gram-positive and Gram-negative pathogens. The antibacterial mechanism appears to be twofold: (1) Membrane disruption through interactions between positively charged CD and bacterial cell walls and (2) metabolic interference via Cur penetration into bacterial cells. This synergistic antibacterial effect is particularly relevant for controlling polymicrobial biofilms characteristic of peri-implantitis.

Given that infection Ctrl is a prerequisite for successful bone regeneration, the potent antibacterial activity of Cur-CD is expected to facilitate subsequent osteogenic processes (Wang et al., 2024). In this study, Cur-CD consistently outperformed both the Cur and Ctrl groups at the imaging, histological, and molecular levels. Micro-CT analysis demonstrated that Cur-CD significantly increased BV/TV, Tb.Th, and Tb.N, indicating enhanced new bone formation and improved trabecular architecture. Histological findings further confirmed the formation of denser and more organized new bone in the Cur-CD group, accompanied by pronounced collagen deposition and mineralized tissue formation, suggesting a shift from fibrous repair to bone regeneration within the defect area (Zheng et al., 2024). At the molecular level, expression levels of Runx2, OPN, and OCN were markedly upregulated in the Cur-CD group, corresponding to the initiation of osteogenic differentiation, matrix production, and mineralization, respectively (Xu et al., 2025). These results collectively indicate that Cur-CD exerts sustained regulatory effects at multiple stages of osteogenesis.

The profound antioxidant capacity of the Cur-CD composite—specifically its ability to mitigate intracellular oxidative stress and restore redox homeostasis—represents the key mechanism rescuing its pro-osteogenic effects. Excessive ROS levels impair osteogenic differentiation and disrupt matrix mineralization, whereas alleviation of oxidative stress can restore osteogenic potential (Sarkar et al., 2022). Previous studies have demonstrated that ROS modulate osteogenesis through signaling pathways, such as Wnt/β-catenin (Zhou et al., 2024). Consistent with our transcriptomic RNA-seq data (Figure 6), GO enrichment significantly enriched the biological term cellular response to oxidative stress, while KEGG analysis identified the AGE-RAGE signaling pathway. AGE-RAGE acts as a typical stress cascade that triggers intracellular ROS overproduction, and accumulated ROS further regulates the Keap1/Nrf2 antioxidant defense system via redox feedback loops. High-level research on curcumin-derived nanocomposites has verified this redox regulatory network: curcumin-based nanoparticles can stabilize Nrf2 and promote its nuclear translocation to induce HO-1 expression, thereby eliminating excess ROS and attenuating oxidative injury in mesenchymal lineage cells. Biomaterial-triggered Nrf2 signaling also upregulates GPX4 to degrade toxic lipid peroxides and block oxidative cell death (Zhou et al., 2022; Kai et al., 2026). Combining our DCFH-DA ROS quantification and transcriptomic profiling with these established research outcomes, we reasonably infer that Cur-CD may restore cellular redox balance through the Keap1/Nrf2/HO-1 cascade and thereby reverse ROS-induced inhibition of osteogenic differentiation. Compared with Cur alone, the superior osteogenic performance of Cur-CD likely arises from the synergistic effects of nanodelivery and material properties. Cur inherently exhibits low aqueous solubility and low bioavailability, thereby impeding the maintenance of effective local concentrations (Lu et al., 2023). Incorporating CD improves its dispersion and stability, while enhancing local retention and cellular uptake. Moreover, CD may contribute to bone regeneration by modulating cellular redox homeostasis or by providing nucleation sites for mineralization (Xia et al., 2020). The enhanced collagen deposition and mineralized bone formation observed in this study further support this hypothesis. However, certain limitations should be noted, including the relatively small sample size and short observation duration, and the exact molecular mechanisms warrant further exploration. Overall, Cur-CD promotes bone regeneration by improving the local microenvironment and orchestrating multiple stages of osteogenesis, highlighting its potential in bone tissue engineering applications.

This dual functionality, promoting bone regeneration while inhibiting bacterial colonization, positions Cur-CD as a promising therapeutic strategy for preventing peri-implantitis and bone tissue engineering. Our composite strategy effectively addresses the inherent limitations of Cur while leveraging the synergistic benefits of CD, offering substantial potential for clinical translation in regenerative medicine.

When contextualizing the phenotypic and transcriptomic observations reported in this preliminary proof-of-concept work, several intrinsic experimental constraints warrant transparent discussion, together with delineated avenues for subsequent mechanistic and translational refinement. At the molecular redox level, our antioxidant characterization is limited to global ROS quantification via DCFH-DA staining; although RNA-seq revealed transcriptional shifts in oxidative stress cascades, definitive validation of the Keap1/Nrf2/HO-1 axis through immunoblotting and pathway inhibition assays is lacking, and dedicated Nrf2 translocation and inhibitor rescue tests will be undertaken to resolve this causal relationship between redox homeostasis and osteogenic recovery. Our antimicrobial profiling is similarly incomplete, confined to aerobic strains relevant to early surgical contamination without assessment of the obligate anaerobes driving chronic peri-implantitis biofilms, though high-impact precedents for curcumin nanomaterials support their broad oral anti-pathogen potential, and dynamic multispecies anaerobic biofilm systems will be utilised to fill this experimental gap in future work (Zhang et al., 2025; Zhang et al., 2026; Xiu et al., 2025; Li et al., 2025). Further restricting translational interpretability is our implant-free, non-infected calvarial defect model, which only captures baseline bone regenerative capacity rather than therapeutic performance against authentic biofilm-associated peri-implant lesions, necessitating the development of complex implant-infected animal models for subsequent *in vivo* validation. Finally, fundamental mechanistic questions surrounding Cur-CD cellular uptake, intracellular trafficking and metabolic turnover fall outside the scope of the current study, forming a distinct line of inquiry to be systematically interrogated in follow-up material–cell interaction research.

## 5 Conclusion

These findings demonstrate that Cur-CD promotes bone regeneration and exerts strong antibacterial activity, with these dual functions laying a preliminary basis for future peri-implantitis research. By enhancing the osteogenic differentiation of rBMSCs and facilitating their migration, Cur-CD demonstrates its potential to mitigate bone loss. Additionally, its dual mechanism against bacterial pathogens, attributed to CD and Cur, effectively combats biofilm formation. This approach not only addresses the solubility challenges of Cur but also suggests a promising strategy for preventive care and management in oral implant procedures, paving the way for further exploration in clinical settings.

## Data Availability

The original contributions presented in the study are publicly available. This data can be found here: https://ncbi.nlm.nih.gov/geo/query/acc.cgi?acc=GSE343587.
