# Supplementary material for: Synthesis of curcumin-derived carbon dots with dual antimicrobial and osteogenic properties for enhanced bone regeneration
Source: Front Cell Dev Biol. 2026 Aug 19;14:1865318. doi: 10.3389/fcell.2026.1865318 (PMC13534087; doi:10.3389/fcell.2026.1865318)
Supplement: Supplementary file 1 [file DataSheet1.docx]

**Supporting Information**

**Synthesis of Curcumin-Derived Carbon Dots with Dual Antimicrobial and Osteogenic Properties for Enhanced Bone Regeneration**

Yu Zhang^1^, Lingyi Kong^1^, Gang Dong^1^, Qingqing Du^1*^

^1^Qilu Medical University, Zibo, China

*Corresponding author: Prof. Dr. Qingqing Du, e-mail: duqingqing@qlmu.edu.cn

**Figure S1. Representative Annexin V-FITC/PI flow cytometry dot plots of rBMSCs incubated with serially diluted Cur-CD.** Nine groups are arranged row-wise: 0.00664, 0.01328, 0.02656, 0.053125, 0.10625, 0.2125, 0.425 0.85 and 1.7 mg/mL Cur-CD. Quadrants Q1–Q4 represent necrotic, late apoptotic, early apoptotic, and viable cells, with the percentage of cells in each quadrant annotated on each panel.

**Figure S2. Evaluation of extracellular matrix mineralization via Alizarin Red S staining.** (A) Whole-well macroscopic Alizarin Red S staining images of rBMSCs after 21 days osteogenic culture. (B) Quantitative absorbance measurement at 560 nm for eluted Alizarin Red S, showing significantly elevated mineral deposition in Cur-CD group. Data = mean ± SD, n=3, **p < 0.01.
